# NCK1-AS1 promotes the progression of melanoma by accelerating cell proliferation and migration via targeting miR-526b-5p/ADAM15 axis

**DOI:** 10.1186/s12935-021-02055-y

**Published:** 2021-07-12

**Authors:** Quan Lin, Yan Jia, Duo Zhang, Hongjuan Jin

**Affiliations:** 1 Department of Plastic and Reconstructive Surgery, The First Hospital of Jilin University, 71 Xinmin Street, Changchun, Jilin China; 2 Department of Obstetrics and Gynecology, The Second Hospital of Jilin University, Changchun, Jilin China

**Keywords:** NCK1-AS1, miR-526b-5p, ADAM15, Proliferation, Migration, Melanoma

## Abstract

**Background:**

Long non-coding RNAs (lncRNAs) are vital regulators of gene expression and cellular processes in multiple cancers, including melanoma. Nevertheless, the function of lncRNA NCK1-antisense 1 (NCK1-AS1) in melanoma remains unknown.

**Methods:**

RT-qPCR was used to analyze the expression of NCK1-AS1, microRNA-526b-5p (miR-526b-5p) and ADAM metallopeptidase domain 15 (ADAM15). Cell proliferation was determined by CCK-8, colony formation and EdU assays. Cell migration was assessed by transwell migration and wound healing assays. Mechanism experiments including luciferase reporter, RIP and RNA pull down assays were conducted to demonstrate the interactions between RNAs. Xenograft model was established to verify the function of NCK1-AS1 and miR-526b-5p in melanoma in vivo.

**Results:**

NCK1-AS1 was overexpressed in melanoma cell lines and NCK1-AS1 knockdown hampers the proliferation and migration of melanoma cells. Besides, miR-526b-5p binds to NCK1-AS1 in melanoma and ADAM15 was validated as its downstream target. Further, the inhibitory effects of NCK1-AS1 knockdown on cell proliferation and migration in melanoma were reversed by the depletion of miR-526b-5p and further counteracted by ADAM15 knockdown. The growth of melanoma tumors was hindered by the down-regulation of NCK1-AS1 or up-regulation of miR-526b-5p.

**Conclusion:**

NCK1-AS1 facilitates cell proliferation and migration in melanoma via targeting miR-526b-5p/ADAM15 axis.

**Supplementary Information:**

The online version contains supplementary material available at 10.1186/s12935-021-02055-y.

## Background

Derived from melanocytes, melanoma is one of the aggressive genres of skin cancer with high morbidity and mortality [1]. Though great progresses have been achieved in melanoma treatment, the survival rate is still dismal [2]. Melanoma presents indistinct symptoms, resulting in most patients are diagnosed at late stage and unable to receive prompt treatment [3]. Nowadays, the major treatment options for patients with melanoma are surgical resection, radiotherapy and combined chemotherapy [4]. However, the results remain unsatisfactory. Hence, it is necessary to explore the underlying mechanism of melanoma to develop better therapies for melanoma patients.

Previous studies have reported that lncRNAs are key regulators in the development of various human cancers. For example, SNHG1 boosts the process of non-small-cell lung cancer via acting as a miR-497 sponge [5]. DILC hinders colorectal cancer cell proliferation and migration [6]. SNHG20 contributes to the progression of breast cancer via sponging miR-495 [7]. Besides, increasing evidence supports that lncRNAs are involved in the development of melanoma. For example, FOXD3-AS1 facilitates the progression of cutaneous malignant melanoma by modulating miR-325 and MAP3K2 [8]. NEAT1 promotes the proliferation, migration, and invasion of melanoma cells by modulating miR-495-3p/E2F3 axis [9]. MEG3 enhances the growth, metastasis and formation of melanoma via regulating miR-21 and E-cadherin [10]. Thus, we intended to further explore lncRNA-related regulation of melanoma.

Moreover, NCK1-AS1 has been proved to be related with the development of different cancers, including prostate cancer [11], osteosarcoma [12], cervical cancer [13], glioma [14], etc. Intriguingly, NCK1-AS1 contributes to the development of cancers by sponging miRNAs as a competing endogenous RNA (ceRNA). For instance, NCK1-AS1 serves as a tumor-propeller in cervical cancer by targeting miR-6857/CDK1 [15]. Therefore, we sought to probe into NCK1-AS1-related regulation of melanoma.

In conclusion, the present study intended to measure the expression level of NCK1-AS1 in melanoma cell lines and further investigate the function and ceRNA mechanism of NCK1-AS1 in melanoma, which might offer insight into the research on NCK1-AS1 in melanoma.

## Materials and methods

### Cell lines culture

Human immortalized keratinocyte HaCaT was procured from Cell Lines Service (CLS) GmbH (Eppelheim, Baden Wurttemberg, Germany) and cultured in high glucose DMEM (Gibco, Grand Island, NY, USA), added with 10% fetal bovine serum (FBS; Gibco) and 1% penicillin–streptomycin (HyClone, Logan, UT, USA). Additionally, human embryonic kidney cells HEK-293 T (ATCC, Manassas, VA, USA) were also incubated in DMEM. Human melanoma cell lines, including A-375 (ATCC), M21 (TongPai Biotechnology Co., Ltd., Shanghai, China), A-875 (China Center for Type Culture Collection, Wuhan, China), A2058 (ATCC) and M14 (TongPai Biotechnology Co., Ltd), were also cultured in DMEM. Cells were cultured in an incubator containing 5% CO_2_ at 37 °C (Additional file 1).

### Real-time quantitative polymerase chain reaction (RT-qPCR)

TRIzol Reagent was added into cell samples to isolate total RNAs, as per the supplier’s protocols (Invitrogen, Carlsbad CA, USA). Afterwards, isolated RNAs were reversely transcribed into cDNA using PrimeScript Reverse Transcriptase Kit (Takara, Shiga, Japan). Then, qPCR was performed with SYBR Green PCR Kit (Takara). The relative expression of each gene was calculated based on the comparative change-in-cycle method (ΔΔCt). U6 or GAPDH was used as internal control. Primers used for RT-qPCR in this study have been listed in Additional file 2: Table S1.

### Cell transfection

Melanoma cells were prepared in 6-well plates. The constructed shRNAs specifically targeting NCK1-AS1 and ADAM15 were purchased from GenePharma (Shanghai, China), along with their negative controls (sh/NCs). Overexpression plasmids of ADAM15 were acquired via inserting the full-length sequence of ADAM15 into pcDNA3.1 vector, and the empty vector pcDNA3.1 served as NC. Additionally, miR-526b-5p inhibitor, miR-526b-5p mimics and corresponding NCs were synthesized (RiboBio, Guangzhou, China). Lipofectamine 3000 (Invitrogen) was used for cell transfection. 48 h later, transfected cells were collected for further study. Over three independent repeats were contained in this assay. The sequence of miR-526b-5p was ACAGAAAGTGCTTCCCTCAAGAG.

### Cell counting kit-8 (CCK-8) assay

5 × 10^3^ melanoma cells were harvested after transfection and seeded in 96-well plates. Then, 10 μL of CCK-8 reagent (Dojindo, Kumamoto, Japan) was added to the transfected cells. Afterwards, the transfected cells were incubated for 2 h. OD value was examined by a spectrophotometer (Thermo Fisher Scientific, Waltham, MA, USA) at 450 nm. More than three independent repeats were performed in this assay.

### EdU assay

EdU assay was conducted in 96-well plates using BeyoClick™ EdU Cell Proliferation Kit (Beyotime, Shanghai, China). After overnight cultivation post transfection, 1  ×  10^4^ transfected melanoma cells were treated with EdU kit for 2 h at 37 °C and DAPI staining solution for 5 min. After rinsing, EdU-positive cells were monitored and photographed via a fluorescence microscope (Olympus, Tokyo, Japan). Over three independent repeats were conducted in this assay.

### Colony formation assay

Transfected melanoma cells were planted into 6-well plates with a seeding density of 600 cells each well and cultured for 14 days. Colonies were defined as aggregates of more than 50 cells. Then, the colonies were fixed by methanol and dyed by 0.5% crystal violet. Finally, the macroscopically visible colonies were manually counted. More than three independent repeats were performed in this assay.

### Transwell migration assay

2  ×  10^4^ transfected melanoma cells were harvested and re-suspended in serum-free medium for transwell migration assay. The transwell upper chambers were purchased from Corning Incorporated (Corning, NY, USA) while lower chamber was supplemented with 100% culture medium. Subsequent to the cultivation for 24 h, the migrated cells into lower chambers were fixed by 4% paraformaldehyde (PFA) and subjected to staining with crystal violet. Five randomly selected fields were analyzed with an Olympus optical microscope (Tokyo, Japan). More than three independent repeats were performed in this assay.

### Wound healing assay

1  ×  10^6^ transfected melanoma cells were planted in 6-well plates until cells reached at about 100% confluence. Then, wounds were scratched with 200-μL pipette tip. The images of wound closure were captured at 0 h and 24 h for the detection of cell migration via ImageJ software (National Institutes of Health, NIH; Maryland, USA). Over three independent repeats were done in this assay.

### Nucleus-cytoplasm fractionation

Cytoplasmic & Nuclear RNA Purification Kit (Norgen, Belmont, CA, USA) was used to isolate nuclear and cytoplasmic RNA fractions. A-375 and M21 cells were treated in cell fractionation buffer, and then subjected to centrifugation. The expression of NCK1-AS1 was detected in nuclear and cytoplasmic RNA fractions via RT-qPCR. U6 and GAPDH were taken as controls. Over three independent repeats were conducted in this assay.

### FISH

The cultured A-375 and M21 cells were prepared to be incubated with FISH probes specifically targeting NCK1-AS1 (RiboBio) in hybridization buffer. Cell nucleus was counterstained by DAPI solution. The fluorescent images were captured using a fluorescence microscope (Olympus). Over three independent repeats were done in this assay. The sequence of NCK1-AS1 FISH probes used in this study has been provided in Additional file 3: Table S2.

### RNA-binding protein immunoprecipitation (RIP)

Melanoma cells were treated with RIP lysis buffer containing protease inhibitors and RNase inhibitors. Subsequently, cell lysates were incubated with the magnetic beads bound with Ago2 antibodies (Anti-Ago2) or IgG antibodies (Anti-IgG) in RIP buffer. 6 h later, the precipitated RNAs were extracted from immunoprecipitates and subjected to RT-qPCR analysis. More than three independent repeats were performed in this assay.

### RNA pull down assay

The melanoma cells were treated with RIPA lysis buffer. Then, cell lysates were mixed with the biotin-tagged probes of NCK1-AS1 or miR-526b-5p and incubated overnight at 4 °C. 30 μL magnetic beads were added after the addition of Deoxyribonuclease I (DNase I). Subsequent to the elution, the RNA-protein mixture was treated with Proteinase K, followed by RNA isolation and RT-qPCR analysis. Over three independent repeats were done in this assay.

### Luciferase reporter assay

Wild-type (WT) or mutant (Mut) sequence of NCK1-AS1 or ADAM15 3′-UTR containing binding site with miR-526b-5p was sub-cloned into pmirGLO dual-luciferase reporter vectors (Promega) to construct luciferase reporter vectors, which were co-transfected into A-375, M21 or HEK-293 T cells with the indicated plasmids. 48 h later, each sample was processed by dual-luciferase reporter assay system (Promega) to visualize and analyze the luciferase activities. More than three independent repeats were performed in this assay.

### Xenograft model

For the animal experiments, 12 female nude mice at the age of 4–6 weeks were commercially bought from the Institute of Zoology, Wuhan University. Mice were infected with melanoma and equally divided for two animal assays. For each assay, three mice in the control group were subcutaneously injected with 10^6^ cells transfected with sh/NC or agomir NC (RiboBio) while the three in the treated group with sh/NCK1-AS1#1 or miR-526b-5p agomir (RiboBio). The tumor volume in mice was detected every 4 days. After 28 days, mice were euthanized. The weight and volume of tumors were measured by an electronic scale and a vernier caliper respectively for analysis. Animal study has been approved by the First Hospital of Jilin University under Grant (No. 2020-0217).

### Statistical analyses

More than three independent repeats were conducted in each experiment and the results were exhibited as means  ±  standard deviation (SD). Student’s t test, one-way ANOVA or two-way ANOVA was applied for the determination of group difference by the use of GraphPad PRISM 6 (GraphPad, San Diego, CA, USA). In addition, data were statistically significant only when p value  <  0.05.

## Results

### NCK1-AS1 is overexpressed in melanoma cells and boosts cell proliferation and migration in melanoma

**Fig. 1:**
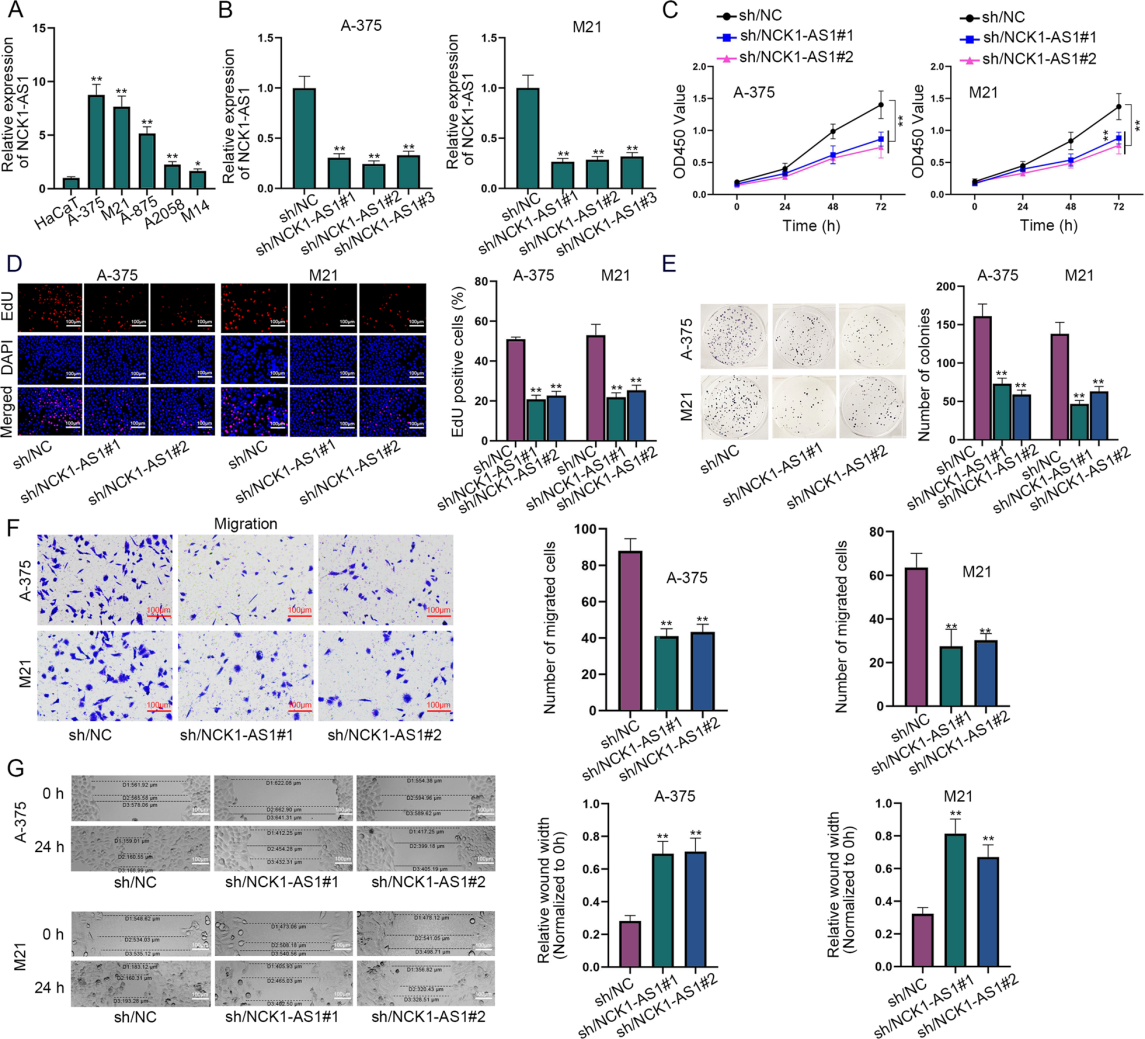
NCK1-AS1 is overexpressed in melanoma cells and boosts cell proliferation and migration in melanoma. **A** RT-qPCR was used to evaluate NCK1-AS1 expression in melanoma cell lines (A-375, M21, A-875, A2085 and M14) and normal control (HaCaT). **B** Knockdown efficiency of sh/NCK1-AS1 in the transfected melanoma cells with sh/NCK1-AS1#1/2/3 was assessed by RT-qPCR. **C**–**E** The proliferative abilities of A-375 and M21 cells after the transfection of sh/NC or sh/NCK1-AS1#1/2 were assessed by CCK-8, colony formation and EdU assays. **F**, **G** The migratory abilities of the transfected melanoma cells with sh/NC or sh/NCK1-AS1#1/2 were examined by transwell migration and wound healing assays. ^*^p  <  0.05, ^**^p  <  0.01

Prior to the exploration of NCK1-AS1’s biological role in melanoma, we applied RT-qPCR to measure NCK1-AS1 expression in melanoma cell lines (A-375, M21, A-875, A2085 and M14) and human immortalized keratinocytes HaCaT. NCK1-AS1 expression was dramatically overexpressed in melanoma cell lines in comparison with that in HaCaT (Fig. 1A). To investigate the loss-of-function or gain-of-function effects of NCK1-AS1 in melanoma, we transfected plasmids of sh/NCK1-AS1#1/2/3 or pcDNA3.1/NCK1-AS1 into melanoma cells to knock down or overexpress NCK1-AS1 (Fig. 1B; Additional file 1: Figure S1A). Among the interference plasmids, we chose sh/NCK1-AS1#1/2 for further studies for their displaying a relatively higher knockdown efficiency. Subsequently, CCK-8, EdU and colony formation assays were performed to evaluate the proliferative ability of melanoma cells. In CCK-8 assay, OD450 value was obviously decreased in the transfected cells with sh/NCK1-AS1#1/2 or increased in the transfected ones with pcDNA3.1/NCK1-AS1 compared with the corresponding control group (Fig. 1C, Additional file 1: Figure S1B). Additionally, the knockdown of NCK1-AS1 severely reduced the percentage of EdU positive cells whereas the up-regulation of NCK1-AS1 distinctly elevated that (Fig. 1D, Additional file 1: Figure S1C). Likewise, the number of colonies was also dramatically reduced after NCK1-AS1 was knocked down but significantly increased after NCK1-AS1 was overexpressed (Fig. 1E, Additional file 1: Figure S1D). Collectively, the above results indicated that NCK1-AS1 knockdown conspicuously inhibits cell proliferation while NCK1-AS1 overexpression has the promoting effect in melanoma.

Furthermore, we conducted transwell and wound healing assays to assess the influence of ectopic expression of NCK1-AS1 on melanoma cell migration. Transwell assays displayed that the number of migrated cells obviously was reduced after the transfection of sh/NCK1-AS1#1/2 into melanoma cells, while the transfection of pcDNA3.1/NCK1-AS1 led to an increase on the number of migrated cells (Fig. 1F, Additional file 1: Figure S1E). Besides, it was implied by wound healing assays that the wound width at 24^th^ h normalized to 0 h was increased by the depletion of NCK1-AS1 but decreased by the up-regulation of NCK1-AS1 relative to controls, suggesting that cell migratory ability was positively regulated by NCK1-AS1 (Fig. 1G, Additional file 1: Figure S1F). In a word, NCK1-AS1 is up-regulated in melanoma cell lines and facilitates cell proliferation and migration in melanoma.

### NCK1-AS1 binds to miR-526b-5p

**Fig. 2:**
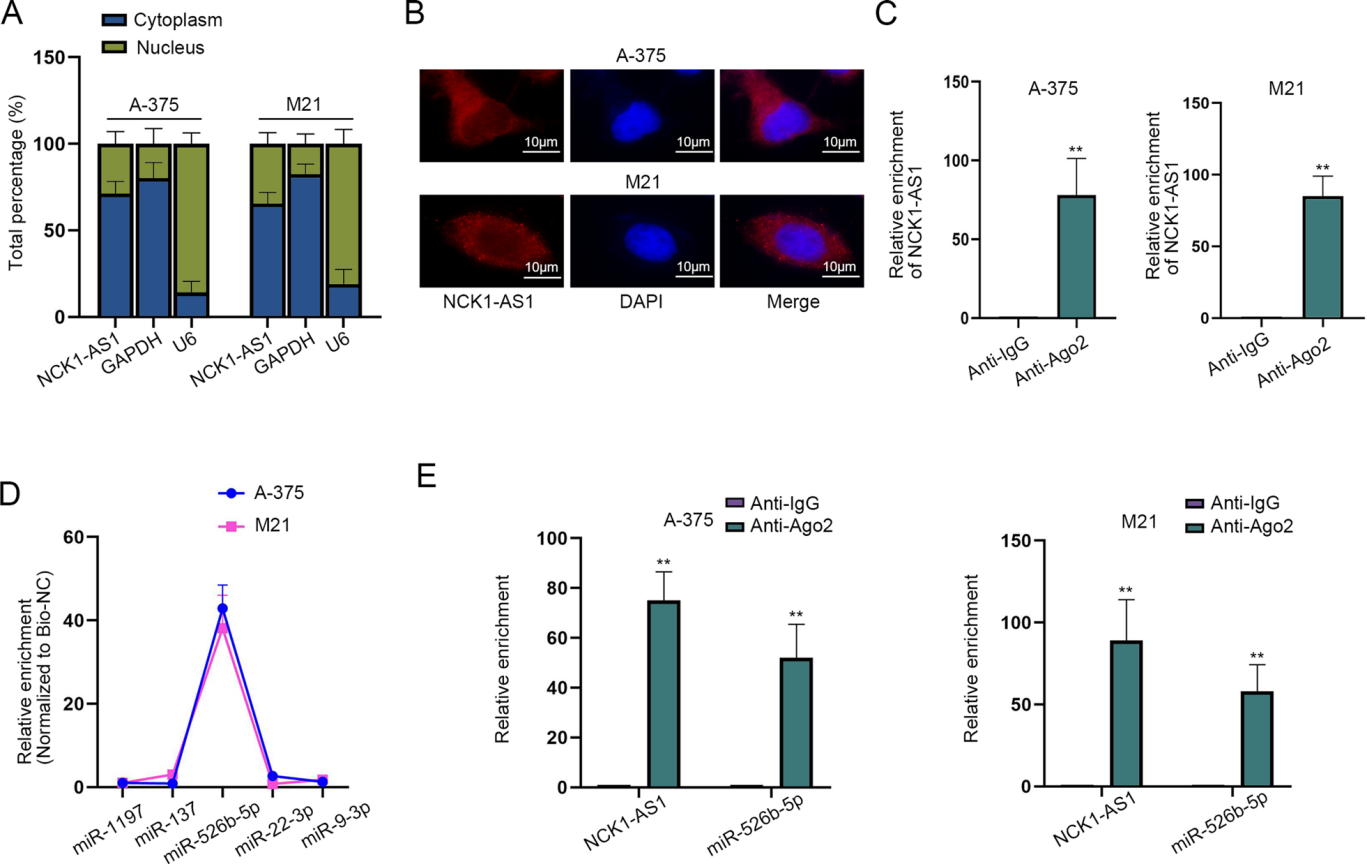
NCK1-AS1 binds to miR-526b-5p. **A**, **B** Subcellular fractionation and FISH assays were conducted to determine the localization of NCK1-AS1 in melanoma cells. **C** RIP assays were performed to examine whether NCK1-AS1 exists in Ago2-RISCs. **D** RNA pull down assays were performed to screen out the downstream gene of NCK1-AS1. **E** RIP assays demonstrated that NCK1-AS1 and miR-526b-5p coexisted in RISCs. ^**^p  <  0.01

Then, we probed into the downstream target of NCK1-AS1. To begin with, subcellular fractionation and FISH assays were performed to locate NCK1-AS1 in melanoma cells. Results disclosed that NCK1-AS1 was mainly distributed in cytoplasm (Fig. 2A, B). Given that ceRNA happens in cytoplasm, NCK1-AS1 was considered to have the potential to be involved in the ceRNA mechanism. Next, RIP assays showed that NCK1-AS1 was abundantly precipitated by Ago2 antibodies compared to Anti-IgG groups (Fig. 2C). Considering that the precipitation was caused by the existence of RNA-induced silencing complexes (RISCs) comprising of Ago2 and microRNAs (miRNAs) [16], it was hypothesized that NCK1-AS1 could bind to specific miRNAs in melanoma cells. Then, starBase (https://starbase.gene.com/; CLIP-Data  ≥  1; Pan-Cancer  ≥  4) was used to predict miRNAs that could bind with NCK1-AS1. Five potential target miRNAs were screened out. RT-qPCR assay showed that the expression of miR-526b-5p in melanoma cells was significantly lower than the other four candidates, implying the potential of miR-526b-5p as the downstream gene of NCK1-AS1 in melanoma (Additional file 1: Figure S1G). Also, RNA pull down assays demonstrated that miR-526b-5p was significantly enriched in Bio-NCK1-AS1 binding pulldowns compared with the other 4 miRNAs (Fig. 2D). In this way, the interaction between NCK1-AS1 and miR-526b-5p was determined. RIP assays showed that miR-526b-5p and NCK1-AS1 both abundantly coexisted in RISCs, verifying that NCK1-AS1 acts as a ceRNA to target miR-526b-5p in melanoma (Fig. 2E). In conclusion, NCK1-AS1 binds to miR-526b-5p as a ceRNA.

### NCK1-AS1 binds to miR-526b-5p to regulate ADAM15

**Fig. 3:**
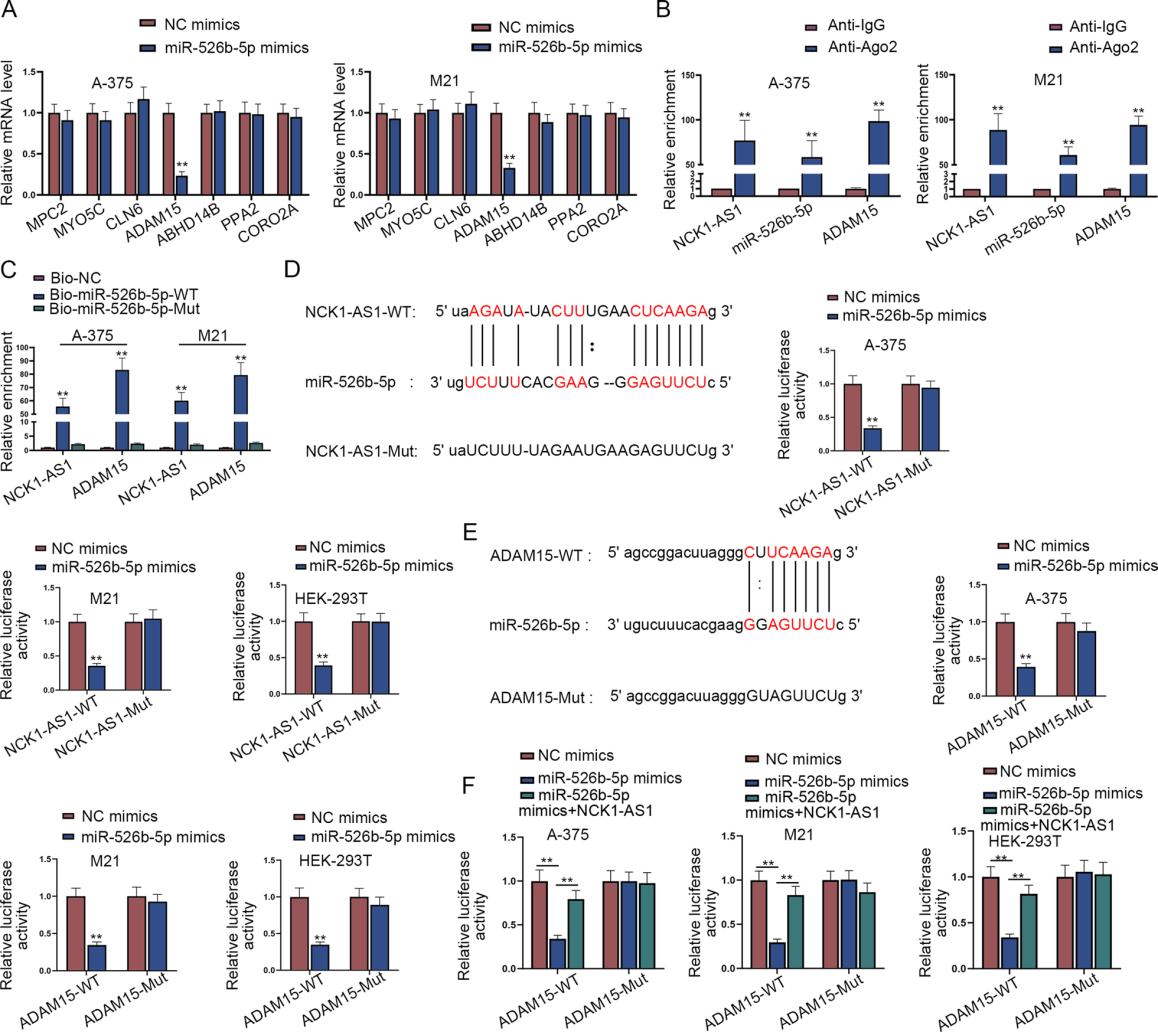
NCK1-AS1 binds to miR-526b-5p to regulate ADAM15. **A** Expressions of 7 mRNAs (MPC2, MYO5C, CLN6, ADAM15, ABHD14B, PPA2 and CORO2A) in the transfected melanoma cells with miR-526b-5p mimics were analyzed by RT-qPCR. **B**, **C** RIP and RNA pull down assays validated the interaction among NCK1-AS1, miR-526b-5p and ADAM15. **D**, **E** The binding sites of miR-526b-5p and NCK1-AS1/ADAM15 were predicted by the starBase. The binding between miR-526b-5p and NCK1-AS1/ADAM15 in melanoma were verified by luciferase reporter assays. **F** Luciferase reporter assays were conducted in A-375, M21, HEK-293 T cells which were transfected with NC mimics, miR-526b-5p mimics or co-transfected with miR-526b-5p mimics and overexpressing NCK1-AS1. ^**^p  <  0.01

Furthermore, we explored the downstream target of miR-526b-5p in melanoma. With the help of bioinformatics starBase (https://starbase.gene.com/; Pan-Cancer  ≥  10), seven putative messenger RNAs (mRNAs) that bind with miR-526b-5p were predicted. RT-qPCR was carried out to evaluate the expressions of candidate mRNAs in melanoma cell lines. ADAM15 was markedly highly-expressed relative to the other seven candidate mRNAs (Additional file 1: Figure S1H). Besides, RT-qPCR was performed to investigate the regulation relationship of miR-526b-5p and candidate mRNAs in melanoma cells. Results revealed that only the level of ADAM15 was terribly diminished by the up-regulation of miR-526b-5p while the other candidate mRNAs had no marked change (Fig. 3A). Thus, ADAM15 was verified as the downstream target of miR-526b-5p in melanoma. Next, RIP assay exhibited that NCK1-AS1, miR-526b-5p and ADAM15 were all significantly enriched in Ago2 antibody groups, suggesting the interplay among miR-526b-5p, NCK1-AS1 and ADAM15 in melanoma cells (Fig. 3B).

Moreover, RNA pull down assays showed that both NCK1-AS1 and ADAM15 were pulled down by Bio-miR-526b-5p-WT while Bio-miR-526b-5p-Mut groups had no marked change compared to controls (Fig. 3C). Then, the binding sites between miR-526b-5p and NCK1-AS1 were predicted prior to luciferase reporter assays. The results of luciferase reporter assays showed that the transfection of miR-526b-5p mimics dramatically weakened the luciferase activity of NCK1-AS1-WT but failed to have the inhibitory effect on that of NCK1-AS1-Mut in A-375, M21 and HEK-293 T cells (Fig. 3D). Accordingly, the luciferase activity of ADAM15-WT was severely decreased while that of ADAM15-Mut was little influenced by the transfection of miR-526b-5p mimics (Fig. 3E). Collectively, the interaction between miR-526b-5p and NCK1-AS1 or ADAM15 was achieved through the corresponding predictive binding site. Moreover, as displayed in rescue experiments, the luciferase activity of ADAM15-WT hindered by the overexpression of miR-526b-5p was reversed by up-regulated NCK1-AS1, verifying the interplay among miR-526b-5p, NCK1-AS1 and ADAM15 (Fig. 3F). To sum up, NCK1-AS1 regulates ADAM15 by binding to miR-526b-5p in melanoma.

### NCK1-AS1 accelerates cell proliferation and migration in melanoma by targeting miR-526b-5p/ADAM15 axis

**Fig. 4:**
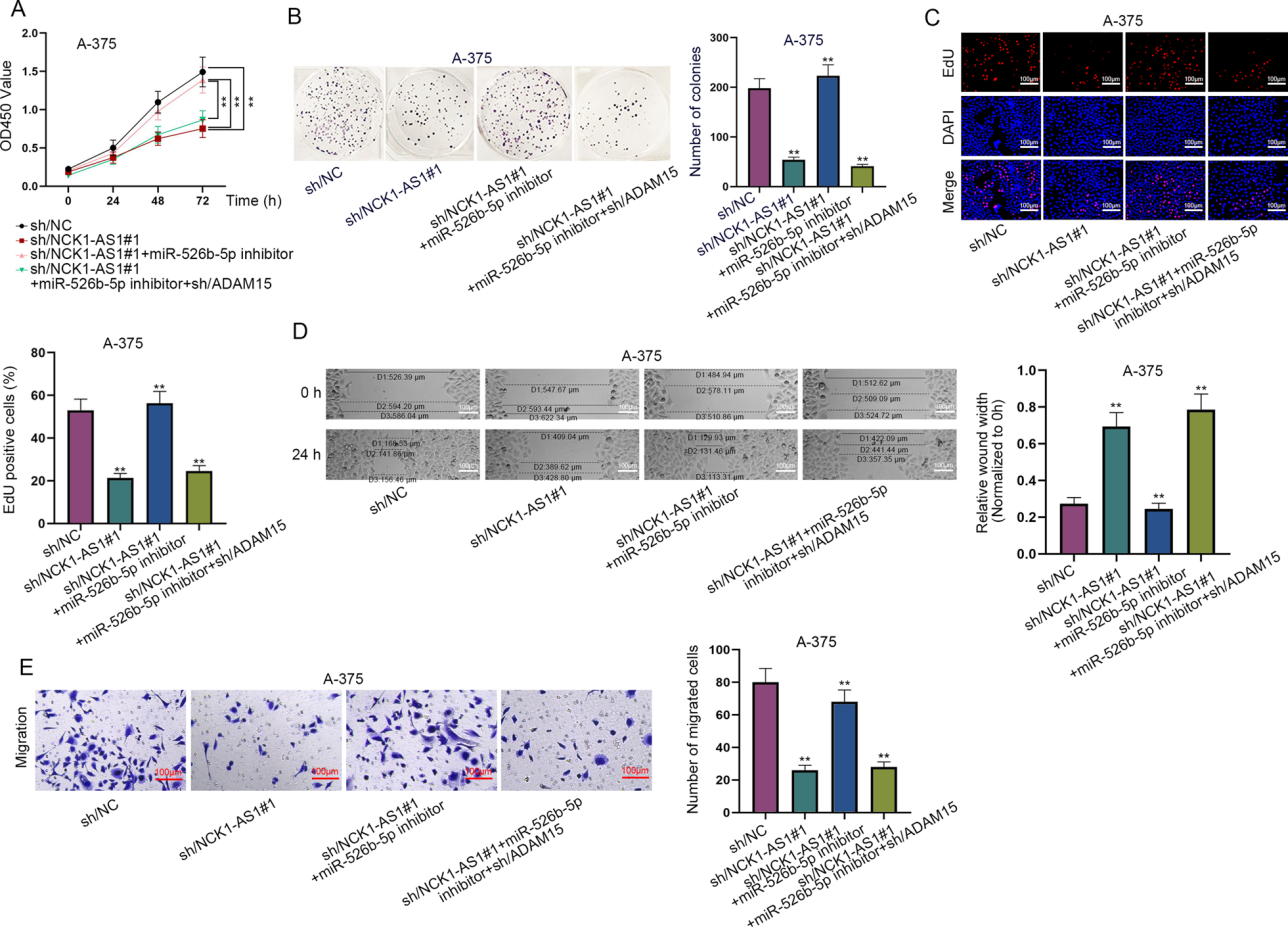
NCK1-AS1 accelerates the proliferation and migration of melanoma cells by targeting miR-526b-5p/ADAM15 axis. **A**–**C** CCK-8, colony formation and EdU assays were used to measure the proliferation of the transfected melanoma cells. **D**, **E** Wound healing and transwell migration assays were carried out to evaluate the migration of the transfected melanoma cells. ^**^p  <  0.01

Next, we carried out rescue experiments to examine whether NCK1-AS1 exerts its function in melanoma cells via regulating miR-526b-5p and ADAM15. Results of CCK-8, colony formation and EdU assays showed that the proliferation of A-375 cells inhibited by NCK1-AS1 knockdown was restored by silenced miR-526b-5p. In addition, ADAM15 knockdown countervailed the promoting effect on cell proliferation induced by miR-526b-5p down-regulation (Fig. 4A–C). Likewise, wound healing and transwell migration assays showed that miR-526b-5p depletion offset the inhibited cell migration imposed by NCK1-AS1 knockdown, but ADAM15 knockdown hampered the migration of melanoma cells again (Fig. 4D, E). Altogether, NCK1-AS1 enhances cell proliferation and migration in melanoma by targeting miR-526b-5p/ADAM15 axis.

### *NCK1-AS1 promotes the carcinogenesis of melanoma while miR-526b-5p has the inhibitory effect *in vivo

**Fig. 5:**
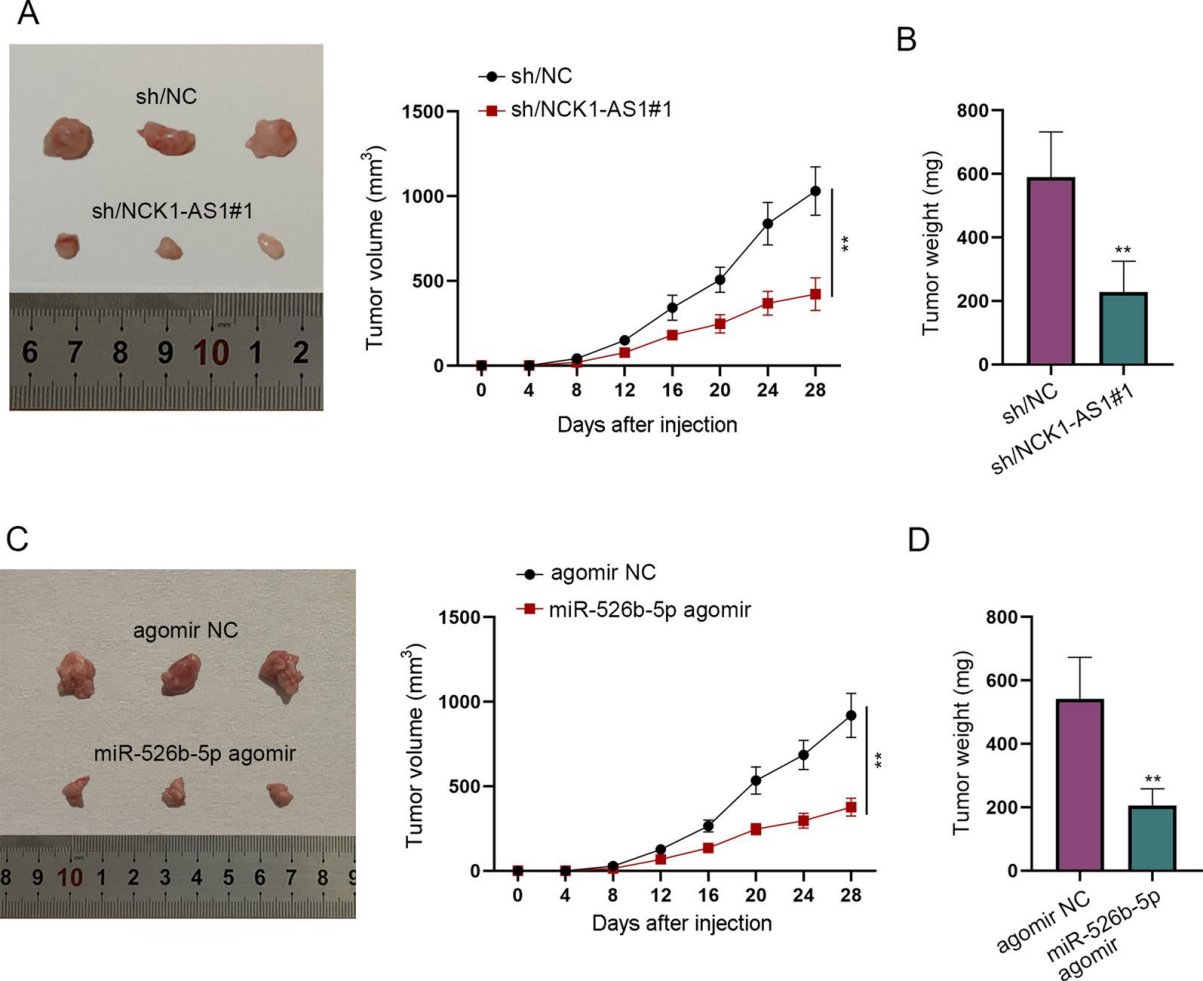
NCK1-AS1 promotes the carcinogenesis of melanoma while miR-526b-5p has the inhibitory effect in vivo. **A** The volume of melanoma tumors in nude mice was detected every four days after the injection of the melanoma cells transfected with sh/NC or sh/NCK1-AS1#1. **B** Tumor weight was evaluated after the excision of tumors from mice. **C** The volume of melanoma tumors in nude mice was detected every four days after the injection of the melanoma cells transfected with agomir NC or miR-526b-5p agomir. **D** Tumor weight was evaluated after the excision of tumors from mice. ^**^p  <  0.01

To verify the hypothesis that NCK1-AS1 functions as a tumor promoter in melanoma and determine the function of miR-526b-5p, xenograft models were established. The tumor growth and weight were significantly impaired by sh/NCK1-AS1#1 (Fig. 5A, B). Expectedly, miR-526b-5p agomir obviously suppressed tumor growth and reduced the tumor weight (Fig. 5C, D). Altogether, NCK1-AS1 drives carcinogenesis in vivo while miR-526b-5p has the opposite effect in melanoma.

## Discussion

Melanoma is a common type of skin cancer which derives from melanocytes and has high morbidity and mortality [1]. In the past decades, emerging evidences have demonstrated that lncRNAs are key regulators in the development of multiple cancers including melanoma. Many lncRNAs have been proved to be oncogenes in melanoma, such as FOXD3-AS1 [8], NEAT1 [9], MALAT1 [17] and MEG3 [10]. Therefore, it is worthwhile to probe into lncRNAs and investigate their functions in melanoma. In this study, NCK1-AS1 was determined as the research object because of its significantly aberrant expression in melanoma cell lines. Based on the previous review, NCK1-AS1 was reported to exert an oncogenic function in the growth of nasopharyngeal carcinoma by sponging miR-135a [18]. Besides, NCK1-AS1 has been validated to accelerate the development of ovarian cancer [19]. In addition, its promoting role in prostate cancer [11], osteosarcoma [12], cervical cancer [13], glioma [14], etc. gives more evidence to the research value of the investigation into NCK1-AS1 in human cancers. In this study, NCK1-AS1 was observed to be overtly overexpressed in melanoma cell lines compared with HaCaT cell line. More importantly, the present study, for the first time demonstrated that NCK1-AS1 serves as an oncogene in melanoma.

In addition, ADAM15 has been proved to be essential in the metastasis of melanoma in previous studies [20, 21]. However, the ceRNA mechanism of ADAM15 in melanoma was not discussed and studied. Herein, we investigated the ceRNA network related to ADAM15 in melanoma. Moreover, miR-526b-5p has been illustrated to inhibit osteosarcoma and oral squamous cell carcinoma [22, 23]. But research on miR-526b-5p in melanoma was still unknown. In this study, the inhibitory effect of miR-526b-5p on the tumorgenesis of melanoma was elucidated through in vivo experiments.

In this study, FISH and subcellular fractionation assays showed that NCK1-AS1 was mainly located in the cytoplasm of melanoma cells. Then, the downstream targets of NCK1-AS1 were determined as miR-526b-5p and ADAM15 through a series of functional assays and mechanism investigations. In this study, NCK1-AS1/miR-526b-5p/ADAM15 axis was validated to modulate the proliferation and migration of melanoma cells. Besides, this study demonstrated that NCK1-AS1 could accelerate the growth of melanoma tumors in vivo.

In short, our study demonstrated the facilitating role of NCK1-AS1 in melanoma and clarified the underlying mechanism of NCK1-AS1/miR-526b-5p/ADAM15 axis in melanoma, offering potential targets for melanoma treatment. As for the future direction, the investigations into the clinicopathological traits of melanoma patients and the mechanism of NCK1-AS1 transcription in melanoma will be performed in the further exploration.

## Conclusion

In conclusion, our study has certified that NCK1-AS1 boosts the progression of melanoma cells via miR-526b-5p/ADAM15 axis, offering insight into melanoma treatment.

## Supplementary Information

**Additional file 1: Figure S1. **(A) Knockdown efficiency of sh/NCK1-AS1#1/2/3 and overexpression efficiency of pcDNA3.1/NCK1-AS1 were assessed by RT-qPCR in the transfected A-875, A2058 and M14 cells. (B–D) The proliferation of A-875, A2058 and M14 cells were assessed by CCK-8, colony formation and EdU assays after the transfection of the indicated plasmids. (E, F) The migration of A-875, A2058 and M14 cells transfected with different plasmids were examined by transwell and wound healing assays. (G) RT-qPCR was performed to analyze relative expression levels of candidate miRNAs in melanoma cells which presented a relatively high expression of NCK1-AS1 (A-375 and M21). (H) RT-qPCR was applied to analyze relative expression levels of candidate mRNAs in A-375 and M21 cells. **P<0.01.

**Additional file 2: Table S1. **Primer sequences used in RT-qPCR.

**Additional file 3: Table S2. **Sequence of NCK1-AS1 FISH probe.

## Data Availability

Not applicable.

## Abbreviations

NCK1-AS: 1NCK1-antisense 1
ADAM15: ADAM metallopeptidase domain 15
lncRNAs: Long non-coding RNAs
mRNAs: Messenger RNAs
miRNAs: MicroRNAs
ceRNA: Competing endogenous RNA
ATCC: American type culture collection
FBS: Fatal bovine serum
qRT-PCR: Quantitative real-time polymerase chain reaction
PFA: Paraformaldehyde
CCK-8: Cell counting kit-8
EdU: 5-Ethynyl-2′-deoxyuridine
DAPI: 4′,6-Diamidino-2-phenylindole
FISH: Fluorescence in situ hybridization
RIP: RNA immunoprecipitation
WT: Wild-type
Mut: Mutant
SD: Standard deviation
ANOVA: Analysis of variance
