# Supplementary figures and images for: NCK1-AS1 promotes the progression of melanoma by accelerating cell proliferation and migration via targeting miR-526b-5p/ADAM15 axis
Source: Cancer Cell Int. 2021 Jul 12;21:367. doi: 10.1186/s12935-021-02055-y (PMC8273965; doi:10.1186/s12935-021-02055-y)

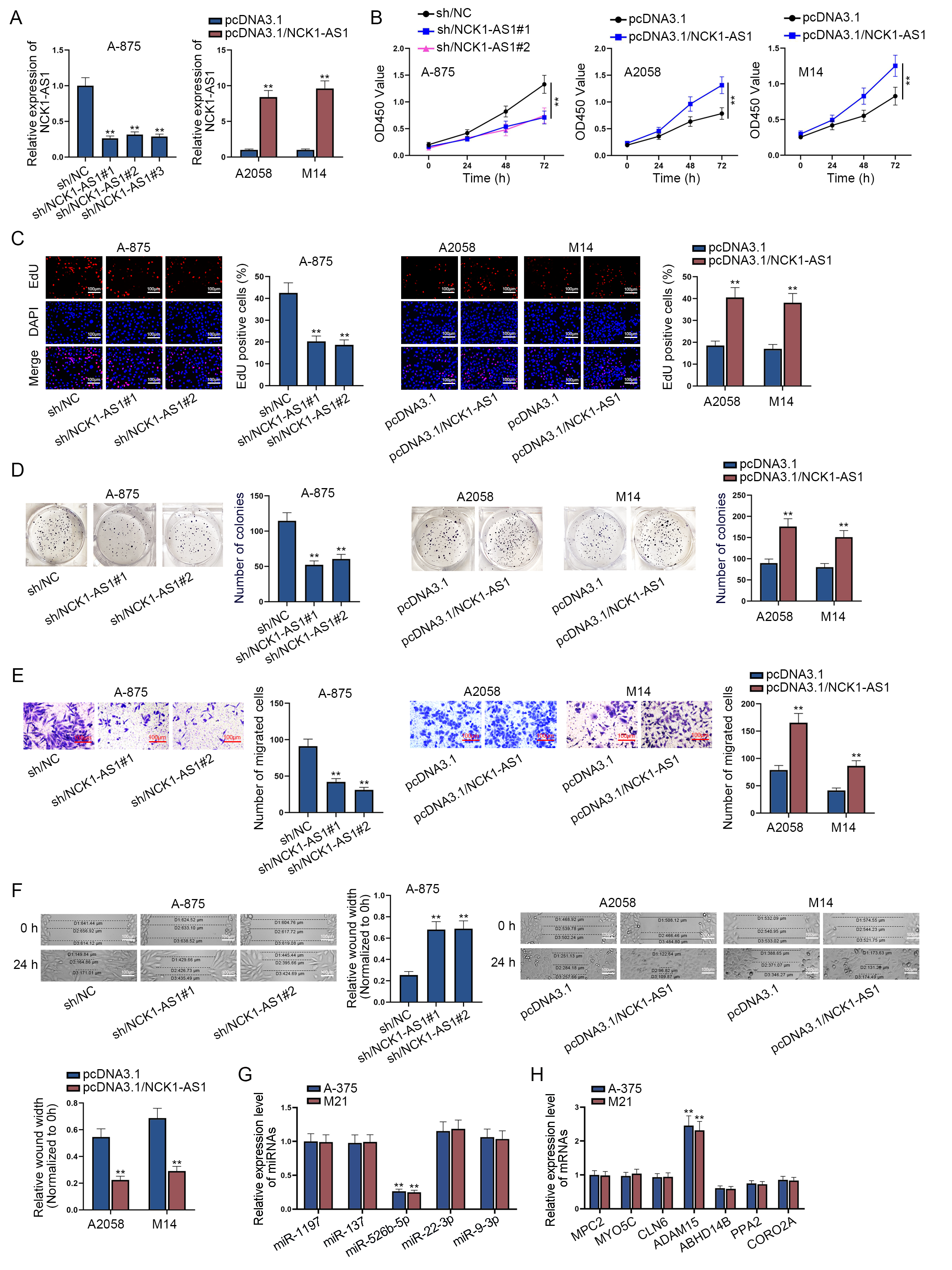

Supplement: Supplementary file 1 — Additional file 1: Figure S1. (A) Knockdown efficiency of sh/NCK1-AS1#1/2/3 and overexpression efficiency of pcDNA3.1/NCK1-AS1 were assessed by RT-qPCR in the transfected A-875, A2058 and M14 cells. (B–D) The proliferation of A-875, A2058 and M14 cells were assessed by CCK-8, colony formation and EdU assays after the transfection of the indicated plasmids. (E, F) The migration of A-875, A2058 and M14 cells transfected with different plasmids were examined by transwell and wound healing assays. (G) RT-qPCR was performed to analyze relative expression levels of candidate miRNAs in melanoma cells which presented a relatively high expression of NCK1-AS1 (A-375 and M21). (H) RT-qPCR was applied to analyze relative expression levels of candidate mRNAs in A-375 and M21 cells. **P<0.01. [file 12935_2021_2055_MOESM1_ESM.tif]
