# Supplementary material for: NCK1-AS1 promotes the progression of melanoma by accelerating cell proliferation and migration via targeting miR-526b-5p/ADAM15 axis
Source: Cancer Cell Int. 2021 Jul 12;21:367. doi: 10.1186/s12935-021-02055-y (PMC8273965; doi:10.1186/s12935-021-02055-y)
Supplement: Supplementary file 2 — Additional file 2: Table S1. Primer sequences used in RT-qPCR. [file 12935_2021_2055_MOESM2_ESM.docx]

**Supplementary Table 1 Primer sequences used in RT-qPCR.**

| **Genes** | **Primer sequences** |
| --- | --- |
| NCK1-AS1 | F: GAGCTTCCCCCTGGTTTCTG R: GCCTGATTCTGGTTAGTCCCT |
| GAPDH | F: GACAGTCAGCCGCATCTTCT R: GCGCCCAATACGACCAAATC |
| U6 | F: TCCCTTCGGGGACATCCG R: AATTTTGGACCATTTCTCGATTTGT |
| miR-1197 | F: TCCTGGTATTTGAAGATGCGGT R: AGTAGACCATGTGTCCTACGTC |
| miR-137 | F: GTCCTCTGACTCTCTTCGGTG R: TGCCGCTGGTACTCTCCTC |
| miR-526b-5p | Sequence: CTCTTGAGGGAAGCACTTTCTGT Stem-loop: CTCAACTGGTGTCGTGGAGTCGGCAATTCAGTTGAGCACAGAA F: TCGCTCTTGAGGGAAGCACT  R: CTCAACTGGTGTCGTGGA |
| miR-22-3p | Sequence: AAGCTGCCAGTTGAAGAACTGT Stem-loop: CTCAACTGGTGTCGTGGAGTCGGCAATTCAGTTGAGCACAGTT F: GCCAAGCTGCCAGTTGAAG  R: CTCAACTGGTGTCGTGGA |
| miR-9-3p | Sequence: ATAAAGCTAGATAACCGAAAGT Stem-loop: CTCAACTGGTGTCGTGGAGTCGGCAATTCAGTTGAGCACTTTC F: GCCGAGATAAAGCTAGATAACC  R: CTCAACTGGTGTCGTGGA |
| MPC2 | F: TTTTCCTCACGTCCCACAAC R: TCCACTTTATCGAGGAGCCG |
| MYO5C | F: GCCGCCAACTCGGGAAC R: ATCGGGAATCCAGACCCTGTT |
| CLN6 | F: CAACGTCATCACGCCCTTTC R: GGGTTCTCACGGACAGACAG |
| ADAM15 | F: GGCTAGTGCTCTCAGCTTCC R: TGAGACTTCGGGCTTCTCAC |
| ABHD14B | F: CTATGTCCTCCTGACCCCAAG R: TCGGAGGAGAAGCGAATACC |
| CORO2A | F: CCAGCCTGGACAAACAATTTAGG  R: AACTGCAATGAAGTGGGGGT |
| PPA2 | F: ATACCTCGGTGGACAAATGCT R: CCAAAGCAGTTCGTGCTCTTAT |

**Note**: F: Forward primer; R: Reverse primer; Stem-loop: Stem-loop RT primer.
