# Supplementary material for: NCK1-AS1 promotes the progression of melanoma by accelerating cell proliferation and migration via targeting miR-526b-5p/ADAM15 axis
Source: Cancer Cell Int. 2021 Jul 12;21:367. doi: 10.1186/s12935-021-02055-y (PMC8273965; doi:10.1186/s12935-021-02055-y)
Supplement: Supplementary file 3 — Additional file 3: Table S2. Sequence of NCK1-AS1 FISH probe. [file 12935_2021_2055_MOESM3_ESM.docx]

**Supplementary Table 2 Sequence of NCK1-AS1 FISH probe.**

| **Plasmids** | **Sequences** |
| --- | --- |
| NCK1-AS1 FISH probes | UUUUUUUUUUUUUUUUUUAAUAAGCAAUCAUUUUAAUACAGGAUUUAAUAAGUUGUCUUAAUAUUUCUGAUAAAUAUUCAGAUGAUUACUUUGAAGUAUUUUGAAAUAAAGAUACUUUCCAUAUUUCAUAAAGCUACUUUUAAUAUAUUAACAUUUAUUGCUCUUGAGUUCAAAGUAUAUCUUAUUCUAGCCACCAAAACAUUUAGAAAAUAGAAAAAAAGGUAAAAACUAAUUUUUUUAUCUCUUAUAUUUCAGACACUUAAUACCCUUACCCCAUUCACUGAAUCCAAUUUCAUUGCUAUUUCCUUGUUUUGUCCUUUUGAGUGGUUCGGGAUCUGCUUCAAAUGACAAAGAGAUGUGAGUAAUAGGGUUCAACGUCUUUCUUAUUUUCAGAUUUAAAGCUCUUCAAUGAUGUAGCUCAAAAUGAAAGUUCUUUCAAUCCAACAACUUUGCAGAGAUGCUGAUCCCUCUCCCAGGUGAAUCCUGGCCAGGCCUUUCUGACAAUCCUGGGAUACCCUGGGGCCAGUAUAGAGUUCAAGGGCAGACCAGACUGGGGGAGGGGUUCCUGAGGGACCUGGAACCAGCAUCACCAGCACAGUCAGAAUUGCAACCCCAUGUGAAUCCUUGUCAGAGGAACUGGAGCCAUGAUACUGGUUUAGAGCACUCGUCUAUUUCUCCCAGGUCCCAAUUUUCAGGUCUGCUUUUCUUUGUUUUUCUUUGUCGCUCCCCGCUCCUAAGAACCUUCGGCUGGGAUGACAUUCUUCAGGUGCAGAAACCAGGGGGAAGCUCCCAGAGCAGUGGGGGCUGAACUCAGGACACAGUCAUUUUUGCUGUUAAGUCUCUCCUGCGGCGCUGAGGAGGCAGGCUCAAAGUAACCAACCAGAGCAGCCGCAGAGGGAAAAGCUAAGACAGUUCUGGUCCGGCCCAGCGCUUCUCACCCUCUAGCUCUCUACUCUCCGCCGCGCAGCCUCCGCUCCCUCCACAUCA |
